# Under the Tech Umbrella: Assessing the Landscape of Telemedicine Innovations (Telemechron Study)

**DOI:** 10.3390/healthcare12060615

**Published:** 2024-03-08

**Authors:** Sandra Morelli, Giuseppe D’Avenio, Carla Daniele, Mauro Grigioni, Daniele Giansanti

**Affiliations:** Centro Nazionale TISP, Istituto Superiore di Sanità, Viale Regina Elena 299, 00161 Roma, Italy; sandra.morelli@iss.it (S.M.); giuseppe.davenio@iss.it (G.D.); carla.daniele@iss.it (C.D.); mauro.grigioni@guest.iss.it (M.G.)

**Keywords:** telemedicine, digital health, technology assessment, digital healthcare

## Abstract

The expanding role of technology assessment in telemedicine is the focus of this study. An umbrella review has been proposed to delve into emerging themes within telemedicine technology assessment by scrutinizing systematic reviews gathered from PubMed and Scopus. The proposed approach was based on a standard narrative checklist and a qualification process. The selection process identified 20 systematic reviews. The main findings underscore the transformative potential of telemedicine, emphasizing technology assessments focused on systematic evaluations, stakeholder engagement, societal impact recognition, targeted interventions, and structured frameworks. While offering valuable insights, the current studies highlight certain limitations that require attention. There is a need for the following: (I) First of all, a more focused approach, primarily centered on a process-centric, multidomain, and generalizable technology assessment (TA). (II) A deeper analysis in specific healthcare areas, including a comprehensive examination of the cost–benefit ratio, peer-to-peer interactions, and a broader inclusion of diagnostic technologies. (III) Greater emphasis on the involved stakeholders, ranging from patients to stakeholders. In conclusion, this study contributes to a comprehensive and nuanced understanding of the continually evolving landscape in telemedicine technology assessment, offering valuable insights for practitioners, researchers, and policymakers alike. Researchers are encouraged to further explore both the established and emerging themes identified in this study.

## 1. Introduction

### 1.1. Background

In the rapidly evolving landscape of healthcare, technological advancements have reshaped disease diagnosis, treatment, and management, offering opportunities for improved quality of life [1,2,3,4,5]. However, the widespread adoption of these technologies introduces challenges and risks, highlighting the critical role of technology assessment. This systematic and multidisciplinary process evaluates the impact of innovations on public health, considering benefits, risks, and costs [2,3]. In healthcare, technology assessment is essential for responsible integration, providing insights into advantages and limitations, guiding clinical and policy decisions, and optimizing resource management [4]. In the dynamic healthcare environment, technology assessment acts as a safeguard, preventing hasty and ineffective adoption and ensuring evidence-based evaluation for enhanced healthcare quality and societal well-being [1,3,5].

Specifically, in telemedicine and digital health, technology assessment is fundamental for evaluating clinical effectiveness, ensuring patient safety, promoting equitable accessibility, and assessing economic sustainability [6,7,8,9,10]. It contributes significantly to evaluating the impact of technological solutions on the diagnosis, treatment, and management of medical conditions [6,7]. Patient safety is prioritized, with the accurate analysis of digital technologies minimizing risks related to privacy, data security, and information accuracy [8]. Equitable access to healthcare technologies is emphasized, and technology assessment plays a crucial role in ensuring innovations are available across diverse populations, preventing disparities in medical care access [6,7,8]. Evaluating the economic sustainability of digital technologies is essential for efficient financial resource allocation, contributing to responsible resource management [9]. Actively involving patients in the assessment process is essential for obtaining valuable insights into usability, user experience, and technology effectiveness [8,9,10]. In summary, technology assessment in telemedicine and digital health ensures a holistic and evidence-based approach, promoting clinical effectiveness, patient safety, equitable accessibility, efficiency, and long-term sustainability [8,9,10]. Moreover, in the regulatory compliance and cybersecurity realm of telemedicine and digital health, technology assessment becomes indispensable [11,12,13]. Attention to regulations, standards, and cybersecurity is paramount to ensuring an ethical, legal, and operational framework for digital health technologies. Technology assessment thoroughly evaluates these elements, playing a crucial role in analyzing compliance with legal and regulatory provisions [12,13]. It prevents potential violations or sanctions, safeguards privacy, and evaluates the effectiveness of implemented security measures to protect sensitive data from potential risks [12]. In an interconnected environment, cybersecurity becomes fundamental, and technology assessment aims to analyze implemented cybersecurity policies and procedures, ensuring the security of networks and data.

In various ways, technology assessment aids in identifying risks and vulnerabilities in digital health technologies, mitigating issues related to security and regulatory compliance [12,13]. It optimizes the allocation of financial resources, preventing investments in technologies that may not meet the required security standards. By providing evidence-based information, technology assessment supports informed decisions regarding the implementation and use of digital health technologies, considering both clinical aspects and those related to security and compliance.

### 1.2. The Rationale for a Narrative Review Study in This Field

Analyses of systematic reviews are valuable for examining emerging and established themes in technology assessment in telemedicine, making them attractive to scholars. Simultaneously, these analyses can also reveal less-explored and less-appealing topics. An umbrella review, which analyzes a set of systematic reviews, provides a comprehensive view of these themes. Two possible approaches are to use a methodology for systematic reviews [14] or to rely on a narrative review methodology [15]. Narrative reviews are preferable when exploring a topic flexibly to obtain a general overview without following a rigorous systematic methodology. They are suitable when aiming to develop conceptual or theoretical frameworks, prioritizing methodological flexibility over an objective synthesis of specific evidence, and seeking a comprehensive overview. When studying emerging topics with a low number of published studies, a systematic review might not be the most suitable choice due to its restrictive study selection.

Technology assessment (TA) in telemedicine is a relatively new field, with a lower volume of publications compared to other themes. For instance, a meticulous yet coherent search on PubMed utilizing a composite key, as reported in Box 1, position 3, led to the identification of only 14 studies dealing with the process of TA in telemedicine [16]. Among these, only six scientific articles [17,18,19,20,21,22] are well focused on the process and encompass a broad range of applications/approaches in technology assessment, not limited to a single domain or a unique telemedicine application. The application of a systematic review approach would lead to further refinement in this selection, posing challenges to obtaining a comprehensive overview. And for this reason, we have chosen to undertake an umbrella review employing a narrative examination of systematic reviews. This strategic approach is aimed at delving deeply into the multifaceted themes within this domain, utilizing a flexible and less rigid analytical tool. This method allows us not only to gain a broad overview but also to capture the intricate nuances associated with emerging themes, thereby enhancing our understanding of the subject matter [15].

Box 1 **The proposed composite keys.**

*(Telemedicine[Title/Abstract]) OR (TeleHealth[Title/Abstract]) OR (Digital health[Title/Abstract]) OR (Digital healthcare[Title/Abstract])*


*((Telemedicine[Title/Abstract]) OR (TeleHealth[Title/Abstract]) OR (Digital health[Title/Abstract]) OR (Digital healthcare[Title/Abstract])) AND (Technology assessment[Title/Abstract])*


*((telemedicine[Title/Abstract]) AND (process[Title/Abstract])) AND (“technology assessment”[Title/Abstract])*

Some of the emerging questions motivating the overview are the following:

- How does technology assessment inform and contribute to the identification of key features for the clinical benefits of telemedicine in healthcare?
- In what ways does technology assessment play a role in enhancing the operational efficiency of healthcare services through telemedicine?
- How is technology assessment utilized to improve the user experience for both patients and healthcare providers in telemedicine?
- What role does technology assessment play in developing strategies to mitigate the risks associated with the implementation of telemedicine?
- How is technology assessment integrated into telemedicine solutions to ensure robust cybersecurity measures and protect sensitive data?

### 1.3. Purpose of the Study

This study is carried out in the framework the Italian National “TELEMECHRON study”(see the funding section for details). The work reported in this study is a section of the activity carried out by the team at Istituto Superiore di Sanità. The study conducts an umbrella review based on a narrative review of technology assessment in telemedicine and digital health to comprehensively understand its impact and the related emerging themes. We aim to analyze multiple systematic review studies, examining how technology assessment influences decision making, guides policy development, and shapes the implementation of innovative technologies in healthcare. The focus is on clarifying the contributions of technology assessment to addressing the benefits, challenges, and regulatory considerations associated with telemedicine and digital health within the evolving healthcare landscape.

## 2. Methods

Performing an umbrella review based on a narrative review of systematic reviews is vital for consolidating evidence and identifying both emerging themes and patterns. It enables a nuanced understanding of the research landscape, an exploration of heterogeneity, and the identification of gaps [23,24].

This umbrella review, based on a narrative review, used the ANDJ standardized checklist designed for narrative reviews [25]. Such a narrative checklist is a methodological tool that provides detailed and structured guidance during the review process. It aids in standardizing the review process by establishing key criteria for use during the analysis, making the process of constructing the study transparent.

The Pubmed and Scopus databases were used in the overview. A qualification methodology was used to choose the studies based on an assessment of qualified parameters [26]. Based on [26], we evaluated each contribution based on six key parameters:

1. Clarity of study rationale in the introduction: This parameter focuses on evaluating how clearly the study’s rationale is presented in the introduction. A well-defined rationale helps readers understand the purpose and context of the research.
2. Appropriateness of work’s design: This parameter assesses whether the chosen design of the study is suitable for addressing the research question. It considers factors such as study type, sampling methods, and data collection techniques.
3. Clarity in describing methods: This parameter examines the clarity of the methodological description. A clear and detailed explanation of the methods allows for the reproducibility of the study and ensures that others can follow and understand the procedures.
4. Clear presentation of results: This parameter focuses on how well the results are presented. This includes the organization of data, the use of figures or tables, and the clarity of language in conveying the study’s findings.
5. Justification and alignment of conclusions with results: This parameter evaluates whether the conclusions drawn from the study are justified by the presented results. It ensures that there is a logical connection between the findings and the overall conclusions.
6. Adequate disclosure of conflicts of interest by authors. This last parameter checks whether the authors have transparently disclosed any potential conflicts of interest. Full disclosure is essential for readers to assess the study’s impartiality and potential biases.

The scoring system involves assigning graded scores (1 = min; 5 = max) to each one of the first five parameters based on the quality of each criterion.

For the last parameter, a binary assessment (Yes/No) is made regarding the disclosure of conflicts.

To preselect studies:

- Each of the first five parameters must obtain a minimum score of 3;
- The last parameter must be marked “Yes” for conflict disclosure.

Only peer-reviewed studies were considered (including congress proceedings if peer-reviewed). 

The defined search query was the following:

(“Search: ((Telemedicine[Title/Abstract]) OR (TeleHealth[Title/Abstract]) OR (Digital health[Title/Abstract]) OR (Digital healthcare[Title/Abstract])) AND (Technology assessment[Title/Abstract])”).

The procedure-based overview identified 20 studies, matching 100% of the PubMed queries [27].

These studies are as follows: [17,28,29,30,31,32,33,34,35,36,37,38,39,40,41,42,43,44,45,46].

## 3. Results

Below is an analysis of the trends of the studies in this field reported in Section 3.1. Following this is a detailed analysis of the key elements emerging from the overview, supported by a table (Table 1) of each study (Section 3.2) and a summary (Section 3.3).

### 3.1. Numerical Trends in Technology Assessment in Telemedicine

**Figure 1:**
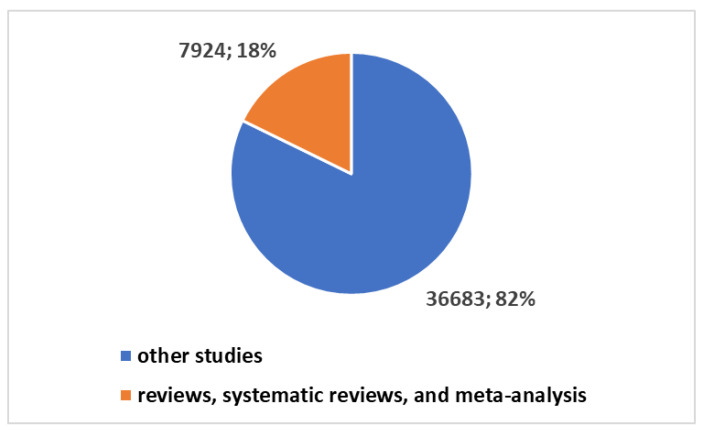
Trends in studies on telemedicine.

**Figure 2:**
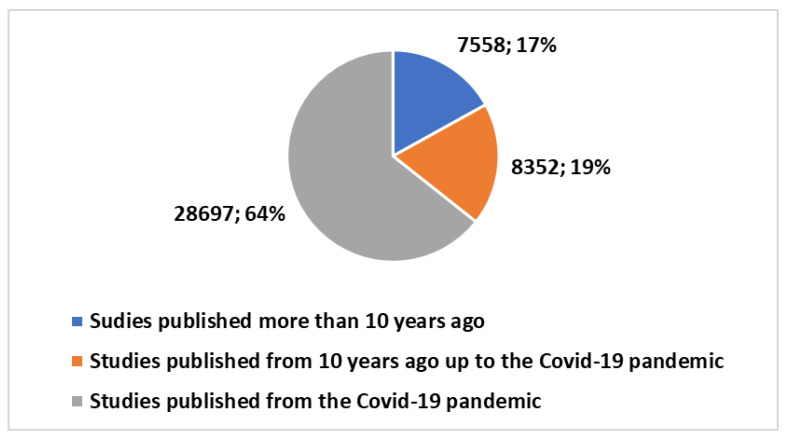
Trends in studies on telemedicine produced over time.

Exploring the trends in scientific publications within the telemedicine domain reveals valuable insights, as highlighted in Box 1. These insights have been extracted and applied in research conducted on the PubMed platform. The cumulative number of publications in telemedicine since 1974 has reached 44,607, as illustrated in Figure 1. Notably, a substantial 83.1% of these publications, totaling 37,049, have emerged in the last decade. Moreover, a staggering 64.3%—equivalent to 28,697 publications—can be attributed to the surge in output following the onset of the COVID-19 pandemic (see Figure 2).

**Figure 3:**
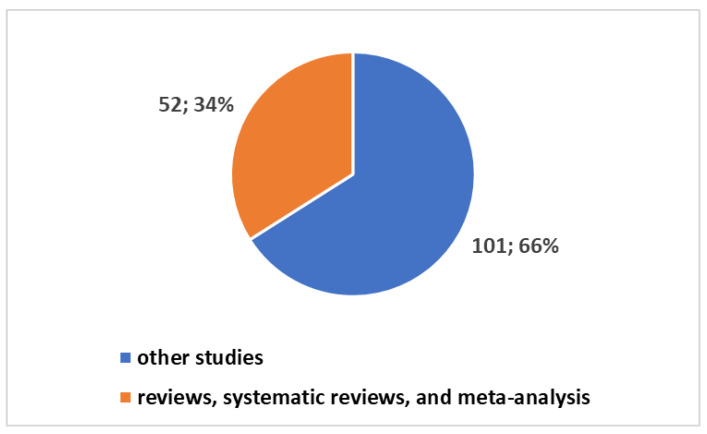
Trends in studies on telemedicine technology assessment.

**Figure 4:**
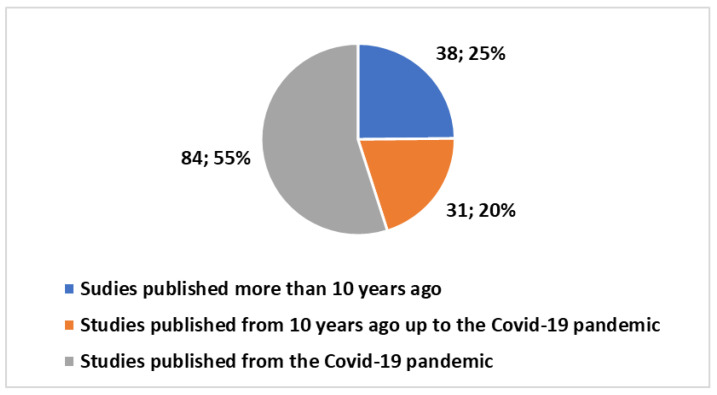
Trends in studies on telemedicine technology assessment produced over time.

Within this landscape, studies addressing technology assessment in telemedicine have amounted to 153 since 1996. Remarkably, these studies constitute a mere 0.34% of the overall research endeavors in the field. Breaking down these 153 studies, 52 fall under the categories of review studies, systematic reviews, and meta-analyses (refer to Figure 3). Zooming in further, 115 of these studies have been conducted in the past decade, with 84 undertaken in the aftermath of the COVID-19 pandemic (Figure 4).

### 3.2. Key Findings

#### 3.2.1. Common Emerging Message

The overviewed studies collectively contribute to a nuanced understanding of the evolving landscape of digital health interventions with a focus on technology assessment, although not always very markedly [17,28,29,30,31,32,33,34,35,36,37,38,39,40,41,42,43,44,45,46]. They emphasize the critical importance of systematically technologically assessing eHealth tools [28], considering TA stakeholder preferences [29], and recognizing the broader impact on well-being [30]. Specific evaluations of TA in some domains, such as the effectiveness of telehealth-based cancer rehabilitation [31] and the integration of tools for peer-to-peer interactions in digital interventions for psychotic disorders [40], shed light on targeted healthcare areas. The application of TA frameworks [32] and innovative approaches, like the “Sandbox Approach” [33], reflects a proactive stance in adapting to the dynamic healthcare technology landscape. Additionally, the focus of TA on cost-effectiveness [35] underscores the need for a comprehensive understanding of the economic implications of digital health interventions. These studies collectively advocate for attention on TA for evidence-based practices, especially in respiratory interventions [42,43,44], diagnostic tools [45], and the longitudinal assessment of telemedicine applications [46], contributing to a holistic view of the strengths and limitations of current digital health evidence.

#### 3.2.2. Key Emerging Themes/Patterns

Delving into greater detail, we can identify the following areas of interest and/or emphasis.

- TA applied eHealth Tool Assessment

Several studies delve into TA applied to eHealth tools, highlighting the importance of evaluating both quality and impact [28,30,32].

- TA and Stakeholder Involvement and Preferences

The consideration of stakeholder preferences in digital health technologies is a recurring theme in TA. Understanding the viewpoints of key stakeholders is seen as crucial for effective resource allocation and strategic planning in the digital health landscape [29].

- Transition to Digital Well-being

A broader perspective on the transition from digital health to digital well-being is explored in TA studies. The studies recognize the evolving nature of healthcare technologies and their impact on overall well-being, extending beyond traditional health metrics, and the importance of the TA in this [30].

- Focus on Specific Health Interventions

Certain studies concentrate their TA on specific health interventions, such as telehealth-based cancer rehabilitation, cardiac rehabilitation, and telemedicine with clinical decision support for critical care [17,31,38,39]. This indicates a targeted approach to assessing the effectiveness of digital interventions in specialized healthcare areas.

- Application of TA Frameworks

The application of technology assessment frameworks is a common thread. Studies highlight the need for structured evaluation frameworks, emphasizing systematic reviews and assessments to analyze the role of digital health in managing chronic diseases [32,34].

- Innovative Approaches to TA solutions

The exploration of innovative approaches, like the “Sandbox Approach” in TA, indicates a recognition of the evolving landscape of healthcare technology assessment. This suggests a proactive stance towards adopting novel methodologies [33].

- Cost-Effectiveness Analysis in TA

Some studies focus on the domain of TA related to the cost-effectiveness of digital health interventions, particularly in managing cardiovascular diseases and supporting severe mental illnesses [35,37]. This demonstrates an awareness of the economic implications and financial viability of these interventions.

- Focus of TA on Peer-to-Peer Interactions in Digital Interventions

The consideration of incorporating peer-to-peer interactions in digital interventions for psychotic disorders highlights the recognition of social interactions as potential contributors to the effectiveness of these technologies and the importance of TA focused on related domains [40].

- TA on Home Telemonitoring Interventions

The critical assessment of systematic reviews and meta-analyses of TA in home telemonitoring interventions underscores the importance of evaluating the methodological quality of existing reviews [41]. This suggests a commitment to understanding the strengths and limitations of current evidence in home telemonitoring for chronic diseases.

- In-depth TA of Specific Respiratory Interventions

Some studies provide in-depth TAs of specific respiratory interventions, such as non-invasive positive pressure ventilation for COPD and pulmonary rehabilitation [42,43,44]. This reflects a detailed exploration of evidence-based practices for managing respiratory conditions.

- TA of Diagnostic Tools

The evaluation of diagnostic tools, such as portable monitoring devices for diagnosing obstructive sleep apnea [45], contributes to the understanding of the evolving landscape of diagnostic technologies in specific healthcare domains.

- TA Longitudinal Perspective on Telemedicine

The inclusion of a systematic review on telemedicine dating back to 2001 [46] suggests a longitudinal perspective, emphasizing the continuous TA of telemedicine applications over time.

Table 1 reports the synthesis of these themes/patterns.

Table 2 reports the key points emerging in the studies.

### 3.3. In-Depth Analysis of the Detected Studies: A Comprehensive Overview

To complement our overview, after having identified the themes and focus elements of the studies, we report here a more far-reaching summary of each individual study.

Jacob et al. [28] explore TA with a focus on the challenges of assessing the quality and impact of eHealth tools in the rapidly evolving landscape of technological advancements. The study systematically analyzes the existing literature, identifying the diverse approaches and criteria used for evaluation. Employing a sociotechnical perspective encompassing technical, social, and organizational factors, the review categorizes 36 unique criteria into 13 clusters. The technical aspects include functionality, content, data management, and design; the social aspects cover human centricity, health outcomes, popularity metrics, and social considerations; and the organizational criteria involve sustainability, scalability, health care organization, context, and developer considerations. The findings emphasize the absence of a standardized framework and advocate for a comprehensive approach that balances these diverse criteria, considering the intricate interplay within the healthcare ecosystem and ensuring sustained user adoption.

Von Huben et al. [29] deal with the importance of TA in investigating stakeholder preferences for digital health technologies (DHTs) in managing chronic diseases, aiming to inform the prioritization of evaluation criteria. Through a best–worst scaling survey involving 1251 participants from diverse stakeholder groups, the research identifies twelve attributes deemed essential, with a focus on safety, technical features, effectiveness, ethics, and economics. Connectedness with the patient’s healthcare team emerges as the most crucial, particularly in facilitating rapid responses to changes in patient care. The study recommends incorporating these prioritized attributes into DHT evaluations, supplemented by a few funder-specific considerations like equity, cost, and system-level implementation.

The systematic scoping review proposed by Smits et al. [30] explores TA in the conceptualization of and approaches to well-being in digital health. The study, based on 117 analyzed papers, reveals that the definition of well-being varies across values like healthy body, functional me, healthy mind, happy me, social me, self-managing me, and external conditions. Design papers emphasize healthy body and self-managing me, while evaluation papers prioritize healthy mind and happy me. Patients with chronic care needs are central users, but user involvement is limited. Both design and evaluation papers focus on providing care support through digital platforms, utilizing different design methods and evaluation approaches. The study suggests the need for multidisciplinary collaborations to optimize digital health for well-being, addressing existing differences between design and evaluation practices.

Brick et al. [31] examine TA in telehealth-based cancer rehabilitation interventions targeting disability in adult cancer survivors. With 68 included studies and 81 unique interventions, the findings reveal that these interventions, mostly post-treatment and lasting an average of 16.5 weeks, demonstrated small effects on disability. The predominant delivery method was telephone calls (59%), administered by nursing professionals (35%), and in a one-on-one format (88%). However, the heterogeneity in disability measurement across studies limits conclusive results. The study underscores the potential of telehealth-based interventions to enhance access to disability-reducing care in cancer rehabilitation, emphasizing the need for more diverse samples, standardized measures, and pragmatic study designs for further research.

Von Huben et al. [32] propose a study investigating trends in primary research on digital health technologies (DHTs) for managing chronic diseases at home, emphasizing the coverage of content crucial for DHT-specific and comprehensive TA. The search identified 178 DHT interventions, primarily randomized controlled trials targeting cardiovascular disease/diabetes in high- to middle-income countries. A content coverage assessment of 112 cardiovascular and diabetes DHT studies revealed that less than half covered DHT-specific content across domains, except for the health problem domain. The study underscores the need for improved trial design and reporting to ensure comprehensive TA and optimal investment decisions in health services.

Leckenby et al. [33] explore the application of regulatory sandboxes in the healthcare sector, initially utilized in financial technologies (FinTech) and later expanding into healthcare. Examining 46 papers and reports, the study identifies four major themes: the history of the regulatory sandbox, a sandbox as a testing environment, a sandbox as a regulatory approach, and examples of sandbox use in healthcare. Although regulatory sandboxes are relatively new in healthcare and primarily employed in high-income countries for digital health technologies, the study suggests their potential application in technology assessment, TA policy, and method development. The transferability of this approach to low- and middle-income countries’ settings should be further assessed.

Von Hubben et al. [34] propose a systematic review aiming to identify and synthesize evaluation frameworks for digital health technologies (DHTs) for managing chronic noncommunicable diseases at home. Examining 44 frameworks, the study reveals a focus on clinical effectiveness and safety issues, with DHT-specific content constituting 28 of the 145 TA Core Model issues. Recognizing the limitations of current TA frameworks for assessing DHTs due to their varied benefit and risk profiles, the study proposes the development of DHT-specific content across all nine TA Core Model domains. Additionally, the researchers plan to create a supplementary evaluation framework for research, HTAs, and the appraisal of completeness for DHTs.

Bonten et al. [35] address the need for structured guidance in TA for eHealth solutions across different study phases. The systematic scoping review identified 57 articles detailing 50 unique evaluation approaches, while a concept mapping study involved 43 eHealth researchers and identified 48 unique approaches. After removing duplicates, a total of 75 unique evaluation approaches were compiled. The researchers developed an “eHealth evaluation cycle” with six study phases and used these phases to compose an “eHealth methodology guide”. This guide, incorporating the 75 evaluation approaches, aims to assist eHealth evaluators in selecting suitable methods for specific study phases and thereby enhance the quality, safety, and successful long-term implementation of eHealth solutions.

Vis et al.’s [36] study aims to support evidence-informed policymaking by identifying frameworks and methods for assessing the impact of eHealth innovations on healthcare. Through a systematic review of the scientific literature from five databases, 21 TA frameworks were identified in 23 articles. The frameworks address various outcomes, including technical performance, functionalities, costs, clinical outcomes, organizational aspects, and system-level considerations. The majority of frameworks fall into dimensional, staged, hybrid, or business modeling categories. However, the study emphasizes the need for standardization in reporting characteristics of eHealth services and specifying assessment outcomes and methods tailored to the functional characteristics of these services to improve the quality and comparability of TAs.

Jiang et al. [37] focus on decision-analytic-model-based studies applying the TA of the cost-effectiveness of digital health interventions (DHIs) in managing cardiovascular diseases (CVDs). A total of 14 studies met the inclusion criteria, with heart failure and stroke being the most frequently addressed CVDs. The studies, published between 2011 and 2018, utilized various technologies, like telemonitoring, video conferencing, and mobile apps. Notably, the DHIs were found to be cost-effective in all the included studies. The quality assessment categorized most studies as good quality, with the majority demonstrating cost-effectiveness, either through cost savings or acceptable incremental cost-effectiveness ratios. This review provides valuable insights into the recent and growing body of evidence supporting the cost-effectiveness of DHIs in CVD management.

Lawes-Wickwar et al. [38] propose a systematic review exploring TA in the applications and efficacy of various telehealth technologies in supporting individuals with severe mental illness (SMI). The review, comprising 29 trials, covers a range of interventions, including computer-assisted cognitive rehabilitation, patient education, web-based self-management, virtual reality, and telephone support. Notably, telephone interventions were found effective in improving medication adherence and reducing symptom severity and inpatient days. Computer-assisted cognitive rehabilitation demonstrated effectiveness in enhancing cognitive function. However, the impact of telehealth on other outcomes varied. The review emphasizes the need for further research to determine the full potential benefits, acceptability, and cost-effectiveness of telehealth for individuals with SMI while acknowledging the limitations posed by the varied quality of the studies reviewed.

Schields et al. [39] propose a study on TA in the domain of economic studies of cardiac rehabilitation (CR) and its components, focusing on full economic evaluations published since 2001. The majority of studies concluded that CR was cost-effective compared to no CR, with incremental cost-effectiveness ratios (ICERs) ranging from USD 1065 to USD 71,755 per quality-adjusted life year (QALY). Specific interventions within CR showed varied results: psychological intervention ranged from dominant (cost-saving and more effective) to USD 226,128 per QALY, telehealth ranged from dominant to USD 588,734 per QALY, and exercise was generally cost-effective. The key drivers of cost-effectiveness included the risk of subsequent events and hospitalization, intervention costs, and utilities. The review provides valuable insights for policymakers, suggesting that CR, especially with exercise as a component, is cost-effective. Further research is needed to determine the most cost-effective design of CR.

Biagianti et al.’s [40] study on TA examines recent digital interventions for individuals with psychotic disorders, focusing on the domain of the feasibility, acceptability, and preliminary efficacy of strategies incorporating online peer-to-peer communication. The search yielded eight publications reporting data from six independent trials with five interventions. The technology supporting peer-to-peer communication varied, including online forums and embedded social networking. Studies with moderated peer-to-peer interactions showed higher retention, engagement, acceptability, and efficacy compared to those without facilitators. Individuals with psychotic disorders actively engaged with moderated peer-to-peer communication and experienced improvements in perceived social support. Involving service users in the intervention design increased acceptability. The findings suggest that individuals with psychotic disorders value and benefit from digital interventions with moderated peer-to-peer interactions, potentially enhancing compliance with other evidence-based therapies.

Mackintosh et al.’s [17] review included two controlled before-and-after studies of TA on the impact of critical care telemedicine on intensive care unit (ICU) and hospital outcomes among adults. Both studies had a high risk of bias. The first study, using a non-randomized stepped-wedge design in seven ICUs, reported a reduction in hospital mortality during the intervention period (an adjusted odds ratio of 0.40). The second study, a non-randomized pre-/post-assessment in 56 adult ICUs, also showed a reduction in hospital mortality (an adjusted hazard ratio of 0.84). The findings suggest a potential benefit of critical care telemedicine, but the overall poor methodological quality highlights the need for high-quality studies to determine the effectiveness and associated costs.

Kitsiou et al. [41] evaluate 24 systematic reviews, including nine meta-analyses, on TA in the domain of the effects of home telemonitoring interventions for patients with chronic diseases. The reviews, published between 1966 and 2012, focused on various chronic diseases, with congestive heart failure being the most studied. The study found that the number of reviews in this area has increased over time. However, many reviews lacked optimal scientific rigor due to methodological issues, and the overall quality did not show improvement. Common shortcomings included insufficient data extraction procedures, manual searches, the inclusion of gray literature, methodological quality assessments of the included studies, and quality of evidence evaluations. The review calls for improved adherence to methodological guidelines in future research on home telemonitoring.

The COPD group proposed a document split into two parts [42,43]. This document outlines a TA conducted by the Medical Advisory Secretariat (MAS) on the effectiveness and cost-effectiveness of noninvasive ventilation (NPPV) for stable chronic obstructive pulmonary disease (COPD) patients. The HTA was initiated in response to a request from the Health System Strategy Division of the Ministry of Health and Long-Term Care. The key details of the assessment are as follows. The objective of the TA was to determine the effectiveness and cost-effectiveness of non-invasive ventilation (NPPV) for stable COPD patients. The clinical need addressed by this TA focused on COPD patients with chronic respiratory failure who may benefit from NPPV. This includes patients with symptoms after optimal therapy, such as hypercapnia or nocturnal hypoventilation, and those frequently hospitalized. Non-invasive positive pressure ventilation (NPPV), specifically bilevel positive airway pressure (BiPAP), was considered. BiPAP involves inspiratory and expiratory pressure, helping to maintain alveolar ventilation and reduce carbon dioxide levels. It is typically used by outpatients at night. The research question addressed in the HTA was the effectiveness and cost-effectiveness of NPPV compared to no ventilation while receiving the usual care for stable COPD patients. The conclusions were based on the quality of evidence, and short-term and long-term studies were considered. In the short-term studies, there was a beneficial effect of NPPV on oxygen and carbon dioxide gas exchange and exercise tolerance. However, the long-term studies showed no significant effects on mortality, lung function, or exercise tolerance. A qualitative assessment suggested a beneficial effect on dyspnea but inconclusive evidence for hospitalizations and health-related quality of life.

Giacomini et al. [44], from the same COPD working group [38], focus on experiences of living and dying with COPD and propose a systematic review and synthesis of the qualitative empirical literature. The analysis provides valuable insights into the domain of acceptance of the diverse experiences of individuals with COPD, their caregivers, and health care providers. Understanding these experiences is crucial for tailoring interventions and support to improve the overall care and well-being of COPD patients.

The objective of the study proposed by Lux et al. [45] was to update a 2002–2003 systematic review of obstructive sleep apnea (OSA) diagnostic testing. The focus was on TA comparing portable sleep testing devices compared to facility-based polysomnography (PSG) in diagnosing OSA and, if portable devices were as effective, determining the relevant sleep and physiologic factors and patient and technician conditions that were important for accurate home testing. The authors concluded that the newer evidence did not significantly alter the earlier findings regarding in-home devices for diagnosing OSA. Different cutoffs for determining OSA for the Apnea–Hypopnea Index (AHI) or Respiratory Disturbance Index (RDI) were used across studies, hindering cross-study comparisons. Sensitivity and specificity values varied, but some studies provided meaningful changes in the probability of OSA. Manual scoring from home monitoring devices correlated better with PSG data than automated scoring. The evidence had mixed internal validity, with the patient populations mainly being male and middle-aged. Importantly, the studies did not evaluate the accuracy of clinical management decisions based on portable results compared to the reference standard.

Roine at al. [46] conducted a systematic review of the telemedicine literature, identifying 1124 studies. Upon closer inspection, 50 TA studies were chosen, with 34 focusing on clinical outcomes and 16 on economic analyses. Most of the literature pertained to pilot projects and had low overall quality. Convincing evidence of effectiveness was found for specific applications like teleradiology, teleneurosurgery, telepsychiatry, and electronic referrals. The economic analyses suggested cost savings, especially in teleradiology. However, the study emphasized limited evidence and recommended only a few telemedicine applications for broader use, emphasizing the need for further research.

## 4. Discussion

The discussion is structured into two parts plus a synoptic scheme, which are editorially translated into three paragraphs.

The initial paragraph, which is Section 4.1, delves into the following: (I) A detailed discussion of the key findings that have emerged in the study results, paying careful attention to the emerging opportunities. (II) An analysis of the limitations and the areas needing a broader investigation.

The second paragraph, which is Section 4.2, deepens the discussion with a focus on the TA process and on what has emerged in the most recent studies. The third section, Section 4.3, reports and discusses a synoptic scheme.

### 4.1. Interpretation of Results: Opportunities, Limitations, and Suggestions for a Brader Investigation

This umbrella review, centered on TA in telemedicine, has spotlighted opportunities, limitations, and recommendations for a broader investigation. The exploration of technology assessment in telemedicine unveils promising prospects for transformative progress in digital health. Emphasizing systematic evaluations, stakeholder engagement, societal impact recognition, targeted interventions, structured frameworks, innovation encouragement, cost-effectiveness focus, and advocacy for evidence-based practices collectively establishes a foundation not only for improving the effectiveness of digital health but also for strategically evolving the landscape. While offering valuable insights, the current studies reveal certain limitations that demand attention. There is a need for more in-depth analyses in specific healthcare areas, a thorough longitudinal assessment of telemedicine applications, broader cost–benefit analyses, an exploration of peer-to-peer interactions, diverse stakeholder representation, enhanced methodological rigor in home telemonitoring studies, a broader inclusion of diagnostic technologies, and a continued expansion of innovative approaches in TA. Looking ahead, the proposed trajectory involves fostering cross-disciplinary collaboration, conducting long-term impact assessments, broadening stakeholder engagement to include diverse perspectives, undertaking comprehensive economic analysis, validating innovative approaches through real-world applications, holistic respiratory intervention assessment, an enhanced evaluation of diagnostic tools, exploring advanced telemedicine applications, prioritizing patient-centric digital solutions, facilitating global comparative analyses, conducting a systematic review of methodological quality, and synthesizing evidence-based guidelines.

Table 3 presents a comprehensive overview, accompanied by pertinent studies, of the discernible patterns and themes that have emerged concerning opportunities in the realm of digital health interventions, as evidenced by systematic review studies. In essence, these studies illuminate pathways for augmenting the effectiveness, stakeholder engagement, societal impact, and economic efficiency of digital health interventions through systematic assessments, targeted approaches, and innovative methodologies. The insights gleaned from the surveyed studies not only contribute to our understanding of the evolving landscape of technology assessment in telemedicine interventions but also underscore the critical significance of systematic evaluation. However, it is imperative to acknowledge the existence of certain limitations, acting as the proverbial flip side of the coin, thereby signaling areas necessitating further refinement, development, and expansion in subsequent research.

Table 4 intricately details the patterns and themes elucidated in systematic review studies pertaining to the limitations that have surfaced. The analytical scrutiny of these limitations not only underscores current challenges but also serves as a roadmap, delineating crucial directions for more extensive investigations in the future.

Table 5 meticulously catalogs the emerging patterns and themes that warrant a more in-depth and comprehensive investigation, as identified in the systematic review studies. These findings underscore areas demanding heightened attention and scrutiny in subsequent research endeavors, serving as catalysts for advancing our understanding of the complex landscape surrounding digital health interventions.

### 4.2. Recent Advancements and Process-Centric Focus in Technology Assessment

#### 4.2.1. Process-Centric Focus in Technology Assessment

Table 3, Table 4 and Table 5 provide a detailed account of all the emerging opportunities, limitations, and areas needing improvements. A significant observation that arises from the analysis in Table 1, Table 2, Table 3, Table 4 and Table 5 is the apparent lack of a specific emphasis on the technology assessment (TA) process when considering the body of systematic review studies as a whole. Only the systematic review conducted in [17] addresses TA in telemedicine with such a focused approach.

Unlike other studies, this review places a pronounced emphasis on TA, emphasizing its critical role and significance in the domain of telemedicine. This singular dedication to TA sets [34] apart, as it delves comprehensively into the processes, methodologies, and specific tools tailored to the unique challenges presented by telemedicine.

This trend could be attributed to both the relatively low percentage of studies explicitly dedicated to TA in this domain, as indicated by the identified trends, and a greater scholarly interest in patterns/themes that are less connected to TA and span multiple domains. Researchers seem to prioritize specificity over the generalizability of methodologies. In a way, this rationale supports the notion, as previously mentioned, of the utility of a more flexible narrative review approach to uncover themes and patterns, especially in areas where the consolidation of medical knowledge is not particularly pronounced. At this juncture, it proves insightful and intriguing to juxtapose the outcomes of the systematic review overview with the results of a more narrowly focused inquiry, one centered specifically on the TA process but inclusive of scientific articles.

This focused investigation, conducted using the composite key outlined in Box 1, position 3, resulted in the identification of only 14 studies [16]. Upon closer examination of the abstracts and focal points, it becomes evident that only five studies [18,19,20,21,22], in addition to the previously analyzed study reported in [17], specifically delve into the processes of technology assessment (TA) within the realm of telemedicine.

The collective narrative emerging from the cited studies underscores a growing recognition and exploration of the potential within the field, placing a strong emphasis on the strategic importance of technology assessment. Diverse in their contexts and methodologies, these studies collectively convey an increasing interest in harnessing telemedicine technologies for clinical decision support and patient-centered care, with a particular focus on the crucial role of utilizing specialized tools and frameworks tailored to the multidimensional nature of TA.

The two studies reported in [18,19] particularly focus on the TA process as a whole, highlighting its strategic and fundamental role in improving telemedicine processes and services. These studies, in conjunction, propose and apply a structured TA methodology based on specific tools/frameworks for a multidomain TA analysis, emphasizing the vital need for tailored instruments in navigating the complexity of telemedicine evaluations.

The study reported in [20] contributes to the evolving landscape of health information technology system evaluations, aligning with the recognized convergence of health informatics and health technology assessment. The author underscores the interconnectedness of these domains, emphasizing the need for comprehensive evaluation approaches and the application of specialized tools to ensure a nuanced understanding of the impact of telemedicine technologies.

The work reported in [21] proposes a technology assessment centered on patient-centered telemedicine pilots in Europe, offering insights based on the MAST approach. This study emphasizes the importance of tailoring technology to meet the specific needs of individuals, providing a patient-centric perspective. Here, the use of specialized tools becomes paramount in ensuring the alignment of technology with individualized patient requirements and preferences.

The study reported in [22] introduces a practical dimension, showcasing a web-based health technology assessment. This experience highlights the application of assessment methodologies in real-world telemedicine projects and emphasizes the evaluation of technology’s impact on specific healthcare domains, leveraging specialized tools within the realm of web technologies.

Overall, these studies, specifically focused on the process, collectively illuminate the ongoing evolution and diverse applications of technology assessment in telemedicine. The resounding message is the increasing significance of systematically evaluating the impact of telemedicine technologies, underscoring the critical role of utilizing tools and frameworks specifically designed for the intricate nature of TA across multifaceted domains. This tailored approach not only enhances the precision of assessments but also contributes to the overall advancement of healthcare practices in the rapidly evolving landscape of telemedicine. Table 6 reports the emerging themes/patterns in these studies focused on the process of telemedicine across multiple domains

#### 4.2.2. Recent Advancements in Technology Assessment

To complete our journey, it is also valuable to compare the outcomes that emerged from the overview of the systematic reviews with the new patterns/themes emerging in studies (not recent reviews).

Examining recent publications unveils evolving trends within this domain that have yet to crystallize into overarching themes, in contrast to the more comprehensive systematic reviews scrutinized in this umbrella review. This endeavor aims to capture the attention of scholars on a large scale, directing focus towards areas that remain in a state of flux and eagerly anticipating in-depth exploration and scrutiny.

An analysis of recent studies within this realm [47,48,49,50,51,52,53,54,55,56,57,58,59,60,61,62,63,64,65,66,67] reveals a diverse tapestry of themes and a wide array of focal points in the field of technology assessment (TA). From a nuanced exploration of the One Health paradigm [47] to considerations of sustainability [49] and the intricate landscape of personalized medicine [50], these studies consistently highlight several key dimensions.

In emphasizing the pivotal role played by stakeholders and the establishment of robust frameworks [52,54,59], these frameworks serve as the scaffolding upon which effective technological assessments can be built, ensuring a holistic understanding of the complex interplay between technology and various stakeholders.

Acknowledging the importance of adopting a collaborative, global approach [56,60] in an era where advancements in technology transcend geographical boundaries, collaborative efforts on an international scale become paramount. This approach fosters the exchange of best practices, accelerates the standardization of assessment methodologies, and facilitates a more unified response to global healthcare challenges.

Consistently stressing the critical need for defining and implementing appropriate metrics, such as key performance indicators (KPIs) [64], these metrics serve as a compass by which the success and impact of digital health solutions can be reliably measured and evaluated.

With a strategic focus on the development of tailored assessment tools designed to navigate the intricacies of digital technologies [48,51,57], these tools are recognized as indispensable assets in ensuring accurate, efficient, and contextually relevant evaluations in the rapidly evolving landscape of digital health.

Noteworthy emphasis on economic impact analyses [55,56] recognizes the profound implications of digital health technologies on healthcare economics, delving into fiscal aspects to unravel the economic dimensions underpinning the adoption and sustainability of such technologies.

Delving into the multifaceted impacts of digital health technologies on social dynamics, behavioral patterns, and the realm of remote rehabilitation [53,65,67], this holistic exploration encompasses societal and behavioral considerations, providing valuable insights into the broader implications of integrating digital interventions into healthcare practices.

Lastly, the pivotal role of medical devices within telemedicine systems is underscored [66]. These devices are recognized as integral components that shape the efficacy and scope of telemedicine initiatives and play a key role in influencing the trajectory of remote healthcare delivery. This further establishes the critical role of technology assessment in shaping the future of healthcare.

From all of this, it emerges that the design of a framework for technology assessment in telemedicine is of paramount importance in the rapidly evolving landscape of healthcare. As telemedicine continues to gain prominence, driven by technological advancements and a growing demand for remote healthcare services, it becomes crucial to establish a systematic and comprehensive approach for evaluating the diverse range of technologies in this domain. Table 7 reports the emerging themes/patterns.

### 4.3. Comprehensive Synoptic Overview

**Figure 5:**
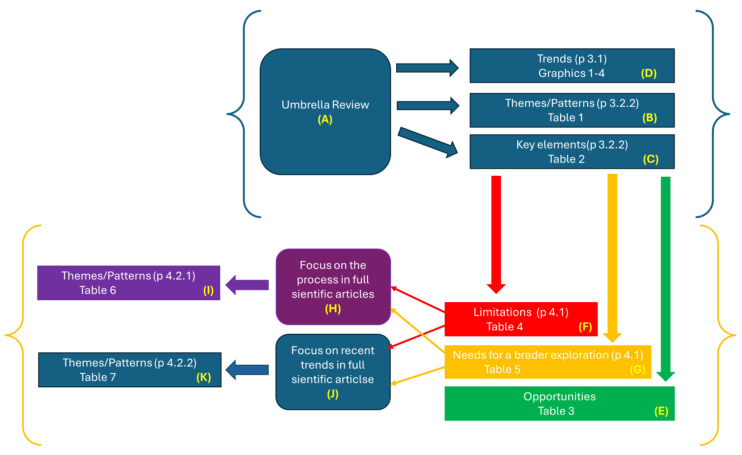
Synoptic diagram representing the flow and the key points of the study.

This extensive investigation conducted via an umbrella review employing a narrative review methodology has enabled the illumination of intricate patterned themes within the realm of telemedicine TA. This comprehensive exploration (Figure 5A) extends beyond mere thematic revelations (Figure 5B,C), encapsulating trends (Figure 5D), nuanced opportunities (Figure 5E), inherent limitations (Figure 5F), and discernible lacunae necessitating in-depth exploration (Figure 5G). The scrutiny of systematic reviews has unveiled a polymorphic approach (Figure 5B,C) within this domain, yet a pervasive constraint persists—a tendency to approach the TA process with a generalized perspective and a multidomain framework. Augmenting the analysis with a focused examination (Figure 5H) of specific studies on the process of TA in multiple domains has brought forth a limited number of studies (Figure 5I) in this domain, coupled with a pressing need to instigate a more substantial impetus.

A closer examination, with a specific focus on the procedural aspects, has unearthed a scarcity of studies in this particular domain, thus underscoring an imperative for heightened research endeavors. The narrative review, characterized by its inherent flexibility and freedom from rigid methodological constraints [15,23,68], has nonetheless adeptly extracted emergent themes and patterns in a robust and consolidated fashion.

Supplementing this analysis with a non-systematic review of recent contributions (Figure 5J) has uncovered additional emerging themes (Figure 5K). Given the contemporary themes identified in recent works and those previously addressed and stabilized, as elucidated in the overview of systematic reviews, a compelling avenue for exploration emerges—the initiation of targeted systematic reviews. In the former scenario, such reviews could prove instrumental in discerning the precise contributions of these identified themes to the corpus of medical knowledge. In the latter, the objective would be to scrutinize potential evolutionary trajectories.

In summation, the overarching overview, coupled with the supplementary analytical discourse, has effectively addressed the pivotal questions arising from the initial hypothesis. It is noteworthy, however, that while a narrative review does not inherently aim at such outcomes, the comprehensive nature of this inquiry contributes substantially to the understanding and advancement of the subject matter at hand.

## 5. Final Takeaway

A telemedicine system is a complex and technologically heterogeneous system [18,19,20,21,22]. Technology assessment emerges as a broad and ever-evolving theme that will inevitably grapple with emerging technological challenges, such as the introduction of artificial intelligence and its integration into medical devices, along with the provision of devices for self-diagnosis directly into the hands of citizens (as seen in teledermatology [69]). In addition to technological challenges of various kinds, it will become increasingly important to systematically address all interconnected domains, from the social and ethical to the economic. The development of methodologies capable of tackling a flexible and adaptable technology assessment, addressing both the technological domain and its interconnected counterparts, is and will be one of the most significant challenges in this field. This holistic approach becomes paramount in navigating the intricate landscape of evolving technologies and their broader implications.

## 6. Limitations of the Study

This study is a narrative review that, through an umbrella review, examines systematic reviews. Two international databases (Pubmed and Scopus) were considered, with one covering a wide range of biomedical topics. Only peer-reviewed studies were included. The narrative review aimed to analyze scientific production, trends, opportunities, limitations, and areas requiring further in-depth exploration. Targeted systematic reviews could be useful for delving into themes identified through the analysis of systematic reviews (e.g., in Section 3.2.2), adopting a living/updating systematic review approach to capture ongoing developments, and exploring additional themes that emerged in the complimentary discussion (e.g., in Section 4.2.2) to identify the state of their consolidation. The study focused exclusively on international productions. Exploring national and local productions through an analysis of technical reports (such as the report in [70], in the case of Italy) and national guidelines could certainly be an interesting avenue for further investigation.

## 7. Conclusions

This umbrella review, focused on telemedicine TA, highlighted opportunities, limitations, and suggestions for a broader investigation. The exploration of technology assessment in telemedicine has illuminated promising opportunities for transformative advancements in digital health. The highlighted systematic evaluations, stakeholder engagement, societal impact recognition, targeted interventions, structured frameworks, innovation encouragement, cost-effectiveness focus, and advocacy for evidence-based practices collectively lay the groundwork for not only improving the effectiveness of digital health but also strategically evolving the landscape.

While offering valuable insights, the current studies highlight certain limitations that require attention. There is a need for the following:

- A more focused approach, primarily centered on a process-centric, multidomain, and generalizable technology assessment (TA).
- A deeper analysis in specific healthcare areas, including a comprehensive examination of the cost–benefit ratio, peer-to-peer interactions, and a broader inclusion of diagnostic technologies.
- Greater emphasis on the involved stakeholders, ranging from patients to stakeholders.

Researchers are encouraged to further explore both the established and emerging themes identified in this study.

## Figures and Tables

**Table 1:** Emerging themes/patterns.

Theme/Pattern	Description	Associated Studies
eHealth TA	Studies focus on evaluating eHealth tools for quality and impact through TA.	[28,29,42]
Stakeholder Involvement and Preferences in TA	Emphasis on considering stakeholder preferences in digital health technology in TA.	[29]
Transition to Digital Well-being in TA	TA exploration of the shift from digital health to digital well-being.	[30]
Specific Health Interventions	TA concentrates on specific health interventions for a targeted assessment.	[17,31,38,39]
Application of TA Frameworks	Common focus on applying technology assessment frameworks systematically.	[32,34]
Innovative Approaches in TA solutions	Exploration of novel approaches like the “Sandbox Approach” in TA.	[33]
Cost-Effectiveness Analysis in TA	Studies assess the TA domain of the cost-effectiveness of digital health interventions.	[35,37]
TA Focus on Peer-to-Peer Interactions in Digital Interventions	Consideration of TA-domain social interactions in digital interventions, emphasizing peer-to-peer aspects.	[40]
TA in Home Telemonitoring Interventions	Critical assessment of TA in home telemonitoring interventions, highlighting the importance of evaluating methodological quality.	[41]
TA In-depth Respiratory Interventions	Studies provide detailed TA of specific respiratory interventions.	[42,43,44]
TA in Diagnostic Tools	TA of diagnostic tools contributes to understanding the evolving landscape of diagnostic technologies.	[45]
TA focus on Longitudinal Perspective on Telemedicine	Inclusion of a systematic review from 2001 suggests a longitudinal perspective on the continuous TA of telemedicine applications.	[46]

**Table 2:** Key elements/points emerging from the overview of systematic reviews.

Review Study	Key Points
Jacob C et al. (2023) [28]	This study delves into the TA of eHealth tools, emphasizing both quality and impact. The researchers employ a systematic literature review and narrative synthesis approach, underscoring the significance of these tools in shaping human factors within the health domain.
von Huben A et al. (2023) [29]	Focusing on stakeholder preferences, this study explores the attributes of digital health technologies that warrant consideration in health service funding. By shedding light on the preferences of key stakeholders involved in the TA, the research contributes valuable insights for the strategic allocation of resources in the digital health landscape.
Smits M et al. (2022) [30]	Addressing the broader transition from digital health to digital well-being, this systematic scoping review provides a comprehensive examination of the increasing importance of TA in this filed. The study reflects the evolving nature of healthcare technologies, acknowledging their impact on overall well-being beyond traditional health metrics.
Brick R et al. (2023) [31]	Focused on telehealth-based cancer rehabilitation interventions, this systematic review scrutinizes their influence on disability. By systematically assessing the existing evidence, the study contributes insights into TA in the domain of the effectiveness of telehealth interventions in the context of cancer rehabilitation, particularly in mitigating disability.
von Huben A et al. (2021) [32]	This study navigates the application of a TA framework to digital health technologies for managing chronic diseases. Conducting a systematic review, the researchers provide an in-depth analysis of the role of digital health in chronic disease management, emphasizing the need for a structured assessment framework.
Leckenby E et al. (2021) [33]	Focused on the “Sandbox Approach” in TA, this literature review explores the potential applications of this approach. The study investigates how the Sandbox Approach could be utilized for evaluating health technologies. It highlights the importance of considering innovative methodologies in the ever-evolving landscape of TA.
Von Huben A et al. (2021) [34]	This systematic review addresses TA for digital technologies in managing chronic diseases. The study emphasizes the need for a systematic evaluation framework, shedding light on the role of digital technologies in chronic disease management. It contributes to the understanding of how such technologies impact healthcare in the context of chronic conditions.
Bonten TN et al. (2020) [35]	Conducted by the eHealth Evaluation Research Group, this study focuses on the development of an “Online Guide for Electronic Health Evaluation Approaches.” The systematic scoping review and concept mapping study aims to provide a comprehensive resource for the TA of electronic health interventions. It underscores the importance of structured approaches in TA applied to assessing the effectiveness of digital health solutions.
Vis C et al. (2020) [36]	This systematic review explores TA frameworks for eHealth. The study emphasizes the need for systematic frameworks to evaluate eHealth technologies, providing insights into existing assessment methodologies. It contributes to establishing a foundation for evaluating the impact and effectiveness of eHealth solutions.
Jiang X et al. (2019) [37]	Focused on TA in the domain of the cost-effectiveness of digital health interventions in managing cardiovascular diseases, this systematic review provides valuable insights. The study assesses the economic aspects of implementing digital health interventions for cardiovascular disease management, contributing to the understanding of the financial implications of these interventions.
Lawes-Wickwar S et al. (2018) [38]	This systematic review evaluates the application of TA to the effectiveness of telehealth in supporting the management of severe mental illnesses. Focusing on a mental health context, the study explores the impact of telehealth interventions on severe mental illness management, providing insights into the effectiveness and application of digital tools in this critical area of healthcare.
Shields GE et al. (2018) [39]	The systematic review investigates the TA of the cost-effectiveness of cardiac rehabilitation. By assessing the economic implications of cardiac rehabilitation programs, the study contributes to understanding the financial aspects associated with these interventions. It emphasizes the importance of evaluating the economic viability of healthcare interventions, specifically in the context of cardiac rehabilitation.
Biagianti B et al. [40]	Focused on psychotic disorders, this systematic review explores the TA domain of the acceptance and potential benefits of incorporating peer-to-peer interactions into digital interventions. The study sheds light on the role of social interactions in digital interventions for psychotic disorders, emphasizing the potential advantages of integrating peer support within these technological solutions.
Mackintosh N et al. (2016) [17]	This systematic review investigates TA in telemedicine with clinical decision support for critical care. By exploring the use of telemedicine in critical care settings, the study provides insights into the potential benefits of integrating clinical decision support systems. It contributes to understanding how telemedicine, coupled with decision support, can enhance critical care practices.
Kitsiou S et al. (2013) [41]	This systematic review focuses on TA in home telemonitoring interventions for patients with chronic diseases. The study critically assesses systematic reviews and meta-analyses. Overall, the study evaluates the methodological quality of existing reviews, offering a comprehensive analysis of home telemonitoring interventions. It contributes to the understanding of the strengths and limitations of current evidence in the field of home telemonitoring for chronic diseases.
COPD Working Group (2012) [42]	This evidence-based analysis, featured in the Ontario Health Technology Assessment Series, focuses on non-invasive positive pressure ventilation for chronic respiratory failure patients with stable chronic obstructive pulmonary disease (COPD). The TA study provides an in-depth analysis of the evidence supporting the use of non-invasive positive pressure ventilation in this specific patient population.
COPD Working Group (2012) [43]	Also published in the Ontario Health Technology Assessment Series, this evidence-based analysis by the COPD Working Group explores pulmonary rehabilitation for patients with chronic pulmonary disease (COPD). The TA study assesses the effectiveness of pulmonary rehabilitation interventions, contributing valuable insights into evidence-based practices for managing COPD.
Giacomini M et al. (2012) [44]	This systematic review and synthesis of qualitative empirical studies, part of the Ontario Health Technology Assessment Series, delves into the experiences of individuals living and dying with COPD. By synthesizing qualitative evidence, the TA study offers a nuanced understanding of the lived experiences of individuals with COPD, contributing to a holistic view of the impact of the disease on patients.
Lux L et al. (2004) [45]	This systematic review, conducted by the Agency for Healthcare Research and Quality (US), provides an update on the TA domain of the effectiveness of portable monitoring devices for diagnosing obstructive sleep apnea. Focusing on diagnostic tools, the study offers insights into the evolving landscape of diagnostic technologies for sleep apnea.
Roine R et al. (2001) [46]	This study, dating back to 2001, contributes to understanding the existing literature on telemedicine, providing insights into the TA of telemedicine applications. Despite its age, this foundational work remains relevant in the context of assessing the evolution of telemedicine over time.

**Table 3:** Opportunities suggested by the overviewed studies.

Opportunities	Description	Associated Studies
Enhance Quality and Impact Evaluation	Standardize TA to ensure the effectiveness and reliability of eHealth tools	[28,30,32]
Consider Stakeholder Preferences	Involve diverse stakeholders in the development and implementation of TA, aligning with their preferences	[29]
Recognize Broader Impact on Well-being	Address societal and cultural implications, acknowledging impacts beyond traditional health metrics in TA	[30]
Targeted Healthcare Interventions	Explore specific areas for TA like cancer rehabilitation and mental health for tailored digital solutions	[40,41]
Application of Health Technology Assessment Frameworks	Implement structured evaluation frameworks (e.g., HTA) for systematic assessment in chronic disease management	[32,34]
Innovative Approaches and “Sandbox Approach”	Encourage innovative methodologies for TA, including the “Sandbox Approach”, for adaptability in the dynamic healthcare technology landscape	[33]
Focus on Cost-Effectiveness	Understand economic implications in the specific TA domain, especially in respiratory conditions, diagnostic tools, and telemedicine applications, for efficient resource allocation	[35,37,41,46]
Advocacy for Evidence-Based Practices	Promote evidence-based practices in TA, particularly in respiratory interventions, diagnostic tools, and telemedicine applications	[42,43,44]

**Table 4:** Concerns/recommendations emerging from the overviewed studies.

Concern/Recommendation	Description	Associated Studies
Limited Depth in Certain Healthcare Areas	The need for more in-depth analyses in specific healthcare areas, such as respiratory interventions beyond COPD, to enhance the understanding of evidence-based practices.	[42,43,44]
Incomplete Longitudinal Assessment	Recognition of the need for a longitudinal perspective, suggesting studies may benefit from more comprehensive and continuous evaluations (a core element for TA) of telemedicine applications for an extended period.	[46]
Scope for More Comprehensive Cost–Benefit Analysis	Emphasis on cost-effectiveness in TA is noted, but there is potential for more comprehensive cost–benefit analyses across a broader spectrum of digital health interventions to provide a holistic view of economic implications.	[35,37,41,46]
Enhanced Exploration of Peer-to-Peer Interactions	There is acknowledgment of peer-to-peer interactions in digital interventions for psychotic disorders in TA, but there is potential for further exploration to understand the dynamics and impact on the effectiveness of these technologies.	[40]
Diversity in Stakeholder Perspectives	Highlighted stakeholder involvement and preferences in TA, suggesting future studies could consider more diverse representation for a comprehensive understanding and enhanced resource allocation in the digital health landscape.	[29]
Methodological Rigor in Home Telemonitoring Studies	Critical assessment of systematic reviews and meta-analyses of home telemonitoring interventions underscores the importance of evaluating methodological quality, indicating a need for continued focus on rigorous methodologies in TA.	[41]
Broader Inclusion of Diagnostic Technologies	Evaluation of diagnostic tools acknowledged in TA, suggesting the need for broader inclusion of different diagnostic technologies in specific healthcare domains for a more comprehensive understanding.	[45]
Expanding Innovative Approaches in Health Technology Assessment (HTA)	Exploration of innovative approaches in TA like the “Sandbox Approach” indicates a proactive stance, suggesting future studies could expand on adopting and developing novel methodologies for HTA to keep up with the continuously evolving healthcare technology landscape.	[32,34]

**Table 5:** Suggestions for advancements emerging from the overviewed studies.

Suggestions for Advancements	Description	Associated Studies
Exploration of Cross-Disciplinary Collaboration	Foster collaboration among healthcare professionals, technologists, and social scientists to enhance interdisciplinary understanding of digital health interventions and their societal implications.	[17,28,29,30,31,32,33,34,35,36,37,38,39,40,41,42,43,44,45,46]
Long-Term Impact Assessment	Conduct longitudinal studies to assess the sustained impact of digital health interventions on patient outcomes, stakeholder engagement, and overall well-being, providing insights into their effectiveness over time.	[30,46]
Inclusive Stakeholder Engagement	Expand stakeholder engagement strategies to include a diverse range of perspectives, such as patients, caregivers, and community representatives, ensuring digital health interventions meet varied population needs.	[36]
Comprehensive Economic Analysis	Conduct in-depth economic analyses, considering not only cost-effectiveness but also broader economic implications, resource allocation strategies, and the potential return on investment for different digital health interventions.	[35]
Validation of Innovative Approaches	Validate and refine innovative methodologies, such as the “Sandbox Approach”, through comparative studies and real-world applications, assessing practicality and effectiveness in adapting to the dynamic healthcare technology landscape.	[33]
Holistic Assessment of Respiratory Interventions	Broaden research on respiratory interventions by conducting comprehensive assessments of various modalities, including in-depth analyses of non-invasive positive pressure ventilation, pulmonary rehabilitation, and emerging technologies.	[42,43,44]
Enhanced Diagnostic Tools Evaluation	Extend the evaluation of diagnostic tools beyond obstructive sleep apnea, exploring a wider range of conditions and technologies for a more comprehensive understanding of the evolving diagnostic landscape in healthcare.	[45]
Advanced Telemedicine Application Studies	Investigate advanced applications of telemedicine, including emerging technologies and novel approaches, to understand their potential in delivering healthcare services, improving patient outcomes, and addressing evolving healthcare challenges.	[46]
Assessment of Patient-Centric Digital Solutions	Prioritize studies that actively involve patients in the co-design and evaluation of digital health solutions, ensuring a patient-centric approach and enhancing user experience and acceptance.	[28,40]
Global Comparative Analyses	Facilitate global comparative analyses by exploring the adoption, effectiveness, and cultural adaptability of digital health interventions across diverse healthcare systems and socio-cultural contexts.	[17,28,29,30,31,32,33,34,35,36,37,38,39,40,41,42,43,44,45,46]
Systematic Review of Methodological Quality	Conduct a systematic review focusing on the methodological quality of existing studies to ensure rigor in study designs, consistency in assessment frameworks, and comparability of results.	[42]
Synthesis of Evidence-Based Guidelines	Synthesize evidence-based guidelines for the responsible integration and deployment of digital health interventions, offering practical recommendations for healthcare practitioners, policymakers, and technology developers.	[17,28,29,30,31,32,33,34,35,36,37,38,39,40,41,42,43,44,45,46]

**Table 6:** Themes/patterns emerging in the studies focused on process-centric telemedicine TA.

Theme/Patterns	Description	Associated Studies
Strategic Role of TA in Telemedicine	The studies emphasize the strategic and fundamental role of the telemedicine TA process in enhancing telemedicine processes and services.	[17,18,19,20,21,22]
Structured TA Methodology	The mentioned studies propose and apply a structured TA methodology, utilizing specific tools and frameworks for a multidomain TA analysis.	[17,18,19,20,21,22]
Tailored Instruments for Complexity	There is a recognition of the vital need for tailored instruments to navigate the complexity of telemedicine TA.	[18,19,20,21,22]
Convergence of Health Informatics and Technology Assessment	There is a recognized convergence of health informatics and health technology assessment, emphasizing the interconnected nature of these domains.	[20]
Patient-Centric Technology Assessment	There is advocacy for a TA centered around patient-centered telemedicine pilots, highlighting the importance of customizing technology to meet individual needs.	[21]
Web-Based Health Technology Assessment	The introduction of a practical dimension with a web-based health technology assessment, emphasizing the application of assessment methodologies in real-world telemedicine projects.	[22]
Emphasis on Specialized Tools	Diffuse emphasis on the design and application of specialized tools in telemedicine TA.	[17,18,19,20,21,22]

**Table 7:** Emerging patterns/themes in recent studies.

Theme/Pattern	Description	Associated Studies
Interconnectedness and Holistic Health	Recognition of the interconnected nature of health, emphasizing collaboration across human, animal, and environmental domains.	[47]
Integrated Digitization for Improved Healthcare	Global trend towards comprehensive digitization in healthcare, with integrated automated-system-level assessment tools for enhancing patient care and improving overall health outcomes.	[48]
Sustainability and Environmental Consciousness	The integration of environmental considerations in digital health technology assessment reflects a global shift towards sustainable healthcare practices.	[49]
Advancements in Personalized Healthcare	Global effort to advance personalized healthcare through behavioral digital biomarkers for real-time monitoring, impacting the management of chronic conditions.	[50]
Dynamic Health Technology Assessment (HTA)	Global challenge in adapting assessment frameworks to the rapidly evolving digital health landscape, focusing on patient-centered evaluation.	[51]
Multistakeholder Value Assessment	Global effort to move beyond purely technological assessments, involving multiple stakeholders in shaping frameworks for diverse perspectives.	[52,54,59]
Global Collaboration in Health Technology	The importance of global collaboration in health technology assessment, with studies exploring international practices and the role of accelerator programs.	[56,60]
Telemedicine’s Impact on Healthcare Delivery	The ongoing impact of telemedicine on global healthcare delivery, highlighted by the design of a Telemedicine Assessment Toolkit (TAT) and studies on teleneurology.	[57,62]
Ethical Dimensions in Digital Health	Global efforts to address ethical considerations in the development of a value framework for patient-facing digital health technologies.	[60]
Standardized Metrics and Key Performance Indicators	Global need for standardized metrics, focusing on the development of key performance indicators (KPIs) for assessing the success and impact of digital health solutions universally.	[64]
Economic Considerations and Reimbursement Models	Exploration of reimbursement processes for Digital Health Applications (DiHA) and practices in public financing, addressing economic considerations in the adoption of digital health technologies.	[65,66]
Telehealth’s Role Beyond the Pandemic	Acknowledgment of telehealth’s role beyond pandemic responses, as seen in the consensus on smart e-Skin Cancer Care in Europe.	[58]
Online Behavioral Intervention in Adolescents	Exploration of online remote behavioral intervention for tics in 9- to 17-year-olds, emphasizing the ORBIT RCT (randomized controlled trial). Comprehensive approach to assessing intervention effectiveness in addressing mental health in adolescents.	[54]
Social Impact of Telemedicine	Evaluation of the social impact of telemedicine in Italy, considering cost opportunities for patients and caregivers, with a focus beyond clinical outcomes.	[65]
Assessment of Medical Device Software	Assessment of medical device software supporting healthcare services for chronic patients in a tertiary hospital, contributing to the ongoing discourse on leveraging technology for chronic disease management.	[66]
State of the Art in Telerehabilitation	Comprehensive overview of the current state of telerehabilitation for musculoskeletal conditions, emphasizing the technology assessment perspective. Valuable resource for healthcare professionals and policymakers aiming to implement digital solutions in rehabilitation.	[67]

## References

[1] Larose H., Lee M., Grueger J., Anota A., Naïditch N., Falissard B., Di Palma M., Chassany O., Khalfallah-Neelz L., Palazuelos-Muñoz S. (2023). Opportunities to improve the adoption of health-related quality of life evidence as part of the French Health Technology Assessment process. Health Res. Policy Syst..

[2] Zhu J., Zhou Y., Wang G. (2023). Cost-Effectiveness of Pharmacist Care in Diabetes Management: A Systematic Review. Diabetes Ther..

[3] Ruscitti P., Feist E., Canon-Garcia V., Rabijns H., Toennessen K., Bartlett C., Gregg E., Miller P., McGonagle D. (2023). Burden of adult-onset Still’s disease: A systematic review of health-related quality of life, utilities, costs and resource use. Semin. Arthritis Rheum..

[4] Sagoo G.S., Robinson T., Coughlan D., Meader N., Rice S., Vale L. (2023). Evaluating high-cost technologies—No need to throw the baby out with the bathwater. Expert Rev. Pharmacoecon. Outcomes Res..

[5] Spiers G., Matthews F.E., Moffatt S., Barker R.O., Jarvis H., Stow D., Kingston A., Hanratty B. (2019). Impact of social care supply on healthcare utilisation by older adults: A systematic review and meta-analysis. Age Ageing.

[6] Mason M., Cho Y., Rayo J., Gong Y., Harris M., Jiang Y. (2022). Technologies for Medication Adherence Monitoring and Technology Assessment Criteria: Narrative Review. JMIR Mhealth Uhealth.

[7] Lappegård K.T., Moe F. (2021). Remote Monitoring of CIEDs-For Both Safety, Economy and Convenience?. Int. J. Environ. Res. Public Health.

[8] Choudhary P., Bellido V., Graner M., Altpeter B., Cicchetti A., Durand-Zaleski I., Kristensen F.B. (2021). The Challenge of Sustainable Access to Telemonitoring Tools for People with Diabetes in Europe: Lessons from COVID-19 and Beyond. Diabetes Ther..

[9] Hilty D., Chan S., Torous J., Luo J., Boland R. (2020). A Framework for Competencies for the Use of Mobile Technologies in Psychiatry and Medicine: Scoping Review. JMIR Mhealth Uhealth.

[10] Graham F., Boland P., Grainger R., Wallace S. (2020). Telehealth delivery of remote assessment of wheelchair and seating needs for adults and children: A scoping review. Disabil. Rehabil..

[11] Saliba V., Legido-Quigley H., Hallik R., Aaviksoo A., Car J., McKee M. (2012). Telemedicine across borders: A systematic review of factors that hinder or support implementation. Int. J. Med. Inform..

[12] Garg V., Brewer J. (2011). Telemedicine security: A systematic review. J. Diabetes Sci. Technol..

[13] Eadie L.H., Seifalian A.M., Davidson B.R. (2003). Telemedicine in surgery. Br. J. Surg..

[14] Denyer D., Tranfield D. (2009). Producing a Systematic Review. The Sage Handbook of Organizational Research Methods.

[15] Sukhera J. (2022). Narrative Reviews: Flexible, Rigorous, and Practical. J. Grad. Med. Educ..

[16] https://pubmed.ncbi.nlm.nih.gov/?term=%28%28telemedicine%5BTitle%2FAbstract%5D%29+AND+%28process%5BTitle%2FAbstract%5D%29%29+AND+%28%22technology+assessment%22%5BTitle%2FAbstract%5D%29&sort=pubdate&size=50.

[17] Mackintosh N., Terblanche M., Maharaj R., Xyrichis A., Franklin K., Keddie J., Larkins E., Maslen A., Skinner J., Newman S. (2016). Telemedicine with clinical decision support for critical care: A systematic review. Syst. Rev..

[18] Giansanti D., Morelli S., Macellari V. (2007). Telemedicine technology assessment part I: Setup and validation of a quality control system. Telemed. J. E Health.

[19] Giansanti D., Morelli S., Macellari V. (2007). Telemedicine technology assessment part II: Tools for a quality control system. Telemed. J. E Health.

[20] Doupi P. (2016). Evolving Health IT Systems Evaluation: The Convergence of Health Informatics and HTA. Stud. Health Technol. Inform..

[21] Ekeland A.G., Grøttland A. (2015). Assessment of mast in European patient-centered telemedicine pilots. Int. J. Technol. Assess. Health Care.

[22] Giansanti D., Morelli S., Maccioni G., Guerriero L., Bedini R., Pepe G., Colombo C., Borghi G., Macellari V. (2009). A web based health technology assessment in tele-echocardiography: The experience within an Italian project. Ann. Ist. Super. Sanita.

[23] Choi G.J., Kang H. (2022). The umbrella review: A useful strategy in the rain of evidence. Korean J. Pain.

[24] Which Review Is That? A Guide to Review Types. https://unimelb.libguides.com/whichreview/umbrellareview.

[25] ANDJ Checklist. https://www.elsevier.com/__data/promis_misc/ANDJ%20Narrative%20Review%20Checklist.pdf.

[26] Giansanti D. (2023). An Umbrella Review of the Fusion of fMRI and AI in Autism. Diagnostics.

[27] https://pubmed.ncbi.nlm.nih.gov/?term=%28%28Telemedicine%5BTitle%2FAbstract%5D%29+OR+%28TeleHealth%5BTitle%2FAbstract%5D%29+OR+%28Digital+health%5BTitle%2FAbstract%5D%29+OR+%28Digital+healthcare%5BTitle%2FAbstract%5D%29%29+AND+%28Technology+assessment%5BTitle%2FAbstract%5D%29&filter=pubt.meta-analysis&filter=pubt.systematicreview&sort=date&size=50.

[28] Jacob C., Lindeque J., Klein A., Ivory C., Heuss S., Peter M.K. (2023). Assessing the Quality and Impact of eHealth Tools: Systematic Literature Review and NarrativeSynthesis. JMIR Hum. Factors.

[29] von Huben A., Howell M., Norris S., Wong K.C., Tang J., Kazi S., Laranjo L., Chow C.K., Howard K. (2023). Stakeholder preferences for attributes of digital health technologies to consider in health service funding. Int. J. Technol. Assess. Health Care.

[30] Smits M., Kim C.M., van Goor H., Ludden G.D.S. (2022). From Digital Health to Digital Well- being: Systematic Scoping Review. J. Med. Internet Res..

[31] Brick R., Padgett L., Jones J., Wood K.C., Pergolotti M., Marshall T.F., Campbell G., Eilers R., Keshavarzi S., Flores A.M. (2023). The influence of telehealth-based cancer rehabilitation interventions on disability: A systematic review. J. Cancer Surviv..

[32] von Huben A., Howell M., Carrello J., Norris S., Wortley S., Ritchie A., Howard K. (2021). Application of a health technology assessment framework to digital health technologies that manage chronic disease: A systematic review. Int. J. Technol. Assess. Health Care.

[33] Leckenby E., Dawoud D., Bouvy J., Jónsson P. (2021). The Sandbox Approach and its Potential for Use in Health Technology Assessment: A Literature Review. Appl. Health Econ. Health Policy.

[34] von Huben A., Howell M., Howard K., Carrello J., Norris S. (2021). Health technology assessment for digital technologies that manage chronic disease: A systematic. Int. J. Technol. Assess. Health Care.

[35] Bonten T.N., Rauwerdink A., Wyatt J.C., Kasteleyn M.J., Witkamp L., Riper H., van Gemert-Pijnen L.J., Cresswell K., Sheikh A., Schijven M.P. (2020). Online Guide for Electronic Health Evaluation Approaches: Systematic Scoping Review and Concept Mapping Study. J. Med. Internet Res..

[36] Vis C., Bührmann L., Riper H., Ossebaard H.C. (2020). Health technology assessment frameworks for eHealth: A systematic review. Int. J. Technol. Assess. Health Care.

[37] Jiang X., Ming W.K., You J.H. (2019). The Cost-Effectiveness of Digital Health Interventions on the Management of Cardiovascular Diseases: Systematic Review. J. Med. Internet Res..

[38] Lawes-Wickwar S., McBain H., Mulligan K. (2018). Application and Effectiveness of Telehealth to Support Severe Mental Illness Management: Systematic Review. JMIR Ment. Health.

[39] Shields G.E., Wells A., Doherty P., Heagerty A., Buck D., Davies L.M. (2018). Cost-effectiveness of cardiac rehabilitation: A systematic review. Heart.

[40] Biagianti B., Quraishi S.H., Schlosser D.A. (2018). Potential Benefits of Incorporating Peer-to-Peer Interactions Into Digital Interventions for Psychotic Disorders: A Systematic Review. Psychiatr. Serv..

[41] Kitsiou S., Paré G., Jaana M. (2013). Systematic reviews and meta-analyses of home telemonitoring interventions for patients with chronic diseases: A critical assessment of their methodological quality. J. Med. Internet Res..

[42] COPD Working Group (2012). Noninvasive positive pressure ventilation for chronic respiratory failure patients with stable chronic obstructive pulmonary disease (COPD): An evidence-based analysis. Ont. Health Technol. Assess. Ser..

[43] COPD Working Group (2012). Pulmonary rehabilitation for patients with chronic pulmonary disease (COPD): An evidence-based analysis. Ont. Health Technol. Assess. Ser..

[44] Giacomini M., DeJean D., Simeonov D., Smith A. (2012). Experiences of living and dying with COPD: A systematic review and synthesis of the qualitative empirical literature. Ont. Health Technol. Assess. Ser..

[45] Lux L., Boehlecke B., Lohr K.N. (2004). Effectiveness of Portable Monitoring Devices for Diagnosing Obstructive Sleep Apnea: Update of a Systematic Review [Internet].

[46] Roine R., Ohinmaa A., Hailey D. (2001). Assessing telemedicine: A systematic review of the literature. CMAJ.

[47] Scott P., Kuziemsky C., Zhu X., Nøhr C., Ammenwerth E., Kukhareva P., Peute L., Marcilly R. (2023). One Health: Insights from Organizational & Social, Technology Assessment and Human Factors Perspectives. Yearb. Med. Inform..

[48] Welzel C., Cotte F., Wekenborg M., Vasey B., McCulloch P., Gilbert S. (2023). Holistic Human-Serving Digitization of Health Care Needs Integrated Automated System-Level Assessment Tools. J. Med. Internet Res..

[49] Alami H., Rivard L., Lehoux P., Ag Ahmed M.A., Fortin J.P., Fleet R. (2023). Integrating environmental considerations in digital health technology assessment and procurement: Stakeholders’ perspectives. Digit Health.

[50] Kolk M., Frodi D.M., Langford J., Meskers C.J., Andersen T.O., Jacobsen P.K., Risum N., Tan H.L., Svendsen J.H., Knops R.E. (2023). Behavioural digital biomarkers enable real-time monitoring of patient-reported outcomes: A substudy of the multicenter, prospective observational SafeHeart study. Eur. Heart J. Qual. Care Clin. Outcomes.

[51] Brönneke J.B., Herr A., Reif S., Stern A.D. (2023). Dynamic HTA for digital health solutions: Opportunities and challenges for patient-centered evaluation. Int. J. Technol. Assess. Health Care.

[52] Pearson S.D., Singh P., Beaudoin F., Campbell J., Schapiro L., Emond S.K., Pearson C. (2023). Institute for Clinical and Economic Review—Peterson Health TechnologyInstitute value assessment framework for digital health technologies. J. Comp. Eff. Res..

[53] Hollis C., Hall C.L., Khan K., Le Novere M., Marston L., Jones R., Hunter R., Brown B.J., Sanderson C., Andrén P. (2023). Online remote behavioural intervention for tics in 9- to 17-year-olds: The ORBIT RCT with embedded process and economic evaluation. Health Technol. Assess..

[54] Main C., Haig M., Chavez D., Kanavos P. (2024). Assessing the Value of Provider-Facing Digital Health Technologies Used in Chronic Disease Management: Toward a Value Framework Based on Multistakeholder Perceptions. Med. Decis. Mak..

[55] Goetz G., Jeindl R., Panteli D., Busse R., Wild C. (2023). Digital Health Applications (DiHA): Approaches to develop a reimbursement process for the statutory health insurance in Austria. Health Policy Technol..

[56] Mezei F., Horváth K., Pálfi M., Lovas K., Ádám I., Túri G. (2023). International practices in health technology assessment and public financing of digital health technologies: Recommendations for Hungary. Front. Public Health.

[57] Agbali R., Andrew Balas E., Heboyan V., Silva J., Coughlin S., Beltrame F., De Leo G. (2023). Design and development of a Telemedicine Assessment Toolkit (TAT) for the assessment of audiovisual telemedicine encounters. J. Telemed. Telecare.

[58] Malvehy J., Dreno B., Barba E., Dirshka T., Fumero E., Greis C., Gupta G., Lacarrubba F., Micali G., Moreno D. (2023). Smart e-Skin Cancer Care in Europe During and after the Covid-19 Pandemic: A Multidisciplinary Expert Consensus. Dermatol. Pract. Concept..

[59] Haig M., Main C., Chávez D., Kanavos P. (2023). A Value Framework to Assess Patient- Facing Digital Health Technologies That Aim to Improve Chronic Disease Management: A Delphi Approach. Value Health.

[60] Njoku C., Green Hofer S., Sathyamoorthy G., Patel N., Potts H.W. (2023). The role of accelerator programmes in supporting the adoption of digital health technologies: A qualitative study of the perspectives of small- and medium-sized enterprises. Digit Health.

[61] Lipprandt M., Klausen A.D., Röhrig R., DESIREE Study Group (2023). Methodology for the Description of Socio-Technical Systems: A Case Study Approach. Stud. Health Technol. Inform..

[62] James J.G., Park J., Oliver A., Xie S.X., Siderowf A., Spindler M., Wechsler L.R., Tropea T.F. (2023). Linked Patient and Provider Impressions of Outpatient Teleneurology Encounters. Neurol. Clin. Pract..

[63] Myung J.E., Strachan L., Shin J., Yim J., Lee S.S. (2023). Reimbursement Coverage Decision Making for Digital Health Technologies in South Korea: Does It Fit the Value Framework Used in Traditional Medical Technologies?. Value Health Reg. Issues.

[64] Brenner M., Weir A., McCann M., Doyle C., Hughes M., Moen A., Ingvar M., Nauwelaerts K., Turk E., McCabe C. (2023). Development of the key performance indicators for digital health interventions: A scoping review. Digit Health.

[65] Papavero S.C., Fracasso A., Ramaglia P., Cicchetti A., de Belvis A.G., Ferrara F.M. (2023). Telemedicine Has a Social Impact: An Italian National Study for the Evaluation of the Cost-Opportunity for Patients and Caregivers and the Measurement of Carbon Emission Savings. Telemed. J. E Health.

[66] Baltaxe E., Hsieh H.W., Roca J., Cano I. (2023). The Assessment of Medical Device Software Supporting Health Care Services for Chronic Patients in a Tertiary Hospital: Overarching Study. J. Med. Internet Res..

[67] Baroni M.P., Jacob M.F.A., Rios W.R., Fandim J.V., Fernandes L.G., Chaves P.I., Fioratti I., Saragiotto B.T. (2023). The state of the art in telerehabilitation for musculoskeletal conditions. Arch. Physiother..

[68] Wong G., Greenhalgh T., Westhorp G., Buckingham J., Pawson R. (2013). RAMESES publication standards: Meta-narrative reviews. BMC Med..

[69] Giansanti D. (2023). Advancing Dermatological Care: A Comprehensive Narrative Review of Tele-Dermatology and mHealth for Bridging Gaps and Expanding Opportunities beyond the COVID-19 Pandemic. Healthcare.

[70] Giansanti G., Morelli S., Bedini R., Macellari V. (2008). Uníesperienza Italiana di Controllo di Qualit‡ in Telemedicina: Il Progetto ERMETE.

